# Characterisation of the Prevailing Multidrug *Pseudomonas aeruginosa* Strains from Surgical Wound Using 16S rRNA Sequencing Technique

**DOI:** 10.21315/mjms2021.28.4.5

**Published:** 2021-08-26

**Authors:** Osagie Aibuedefe Eremwanarue, Stanley Udogadi Nwawuba, Olalekan Hakeem Shittu

**Affiliations:** 1 Department of Plant Biology and Biotechnology, University of Benin, Ugbowo, Benin City, Nigeria; 2 Lahor Research Laboratories and Diagnostics Centre, Benin City, Nigeria; 3 Centre for Forensic Programmes and DNA Studies, University of Benin, Ugbowo, Benin City, Nigeria

**Keywords:** 16S rRNA sequencing, molecular characterisation, Pseudomonas aeruginosa, wound infection, nosocomial infections

## Abstract

**Background:**

*Pseudomonas aeruginosa* (*P. aeruginosa*) is prevalent in hospital-acquired surgical wound infections. It exhibits both innate and acquired resistance to a broad range of antimicrobials and remains a principal problem in clinical practice.

**Methods:**

In total, 284 sterile surgical wound swabs (142 each) were collected from two government hospitals: Central Hospital Benin (CHB) and University of Benin Teaching Hospital (UBTH) in Benin City, Nigeria. *Pseudomonas* spp. isolated from both hospitals were screened with eight different antibiotics by way of disk diffusion method. Polymerase chain reaction (PCR) amplification of 34 multiple drug-resistant isolates was carried out using genus-specific primer set on extracted genomic DNA for the identification of *Pseudomonas* spp. and substituent 16S rRNA sequencing to determine the prevailing strains in the two locations.

**Results:**

Sixty-two *Pseudomonas* spp. were isolated from the two locations (27 isolates from CHB and 35 isolates from the UBTH). Surgical wound infections screened with regularly used antibiotics revealed that 17 (62.9%) isolates from CHB and 20 (57.1%) isolates from UBTH were multiple drug resistant *Pseudomonas* spp. PCR identification using *Pseudomonas* spp. specific primer showed that 16 (94.1%) isolates from CHB and 18 (90%) isolates from UBTH were confirmed. The 16S DNA sequencing revealed that *P. aeruginosa* strain H25883 was dominant in both locations.

**Conclusion:**

High antibiotic resistance among *P. aeruginosa* isolates was established in our study. PCR technique revealed a more reliable method of bacterial identification. H25883 strain of *P. aeruginosa* is the prevalent strain in both locations and it should be given attention in nosocomial surgical wound infections.

## Introduction

Post-surgical wound infection is the major source of nosocomial infection in surgical patients, accounting for 39.9% of all infections. It mainly causes post-operative morbidity, resulting in longer hospital stay, increased hospital bill and incidences of postoperative death. Generally, wound infections are a result of wound contamination caused by endogenous bacteria from the patient’s skin, mucous membrane or hollow viscera (1). The development of an infection in any wound is subjective largely to the virulent nature of the microorganism and immunity of the patient. Nevertheless, when pus oozes from a closed surgical opening along with signs of inflammation in the adjoining tissues, it is referred to as wound infection (2, 3).

*Pseudomonas aeruginosa (P. aeruginosa*) is a gram-negative bacterium. It is non-sporous, motile and a facultative anaerobe. It is accountable for a wide range of diseases in both humans and animals (4). Generally, *P. aeruginosa* is an opportunistic and nosocomial infectious organism that could develop infections in burns, injury, surgical wounds and in immunocompromised subjects (5, 6). Incidences of *P. aeruginosa* infections are on the rise worldwide due to their mechanisms of survival, adaptation and resistance to different types of antibiotics (7). Wound infection caused by *P. aeruginosa* is considered a major cause of morbidity and mortality (8). The immense use of routine broad-spectrum antibiotics has increased the resistance of *P. aeruginosa* to clinical drugs, which has led to serious therapeutic problems (9). Thus, timely and precise diagnosis is essential for proper treatment and also to control future disease outbreaks. A wide range of diagnostic methods have been established for *P. aeruginosa* identification. They include phenotypic methods (10), electrochemical techniques (11) such as enzyme-linked immunosorbent assay (12), and molecular methods such as polimerase chain reaction (PCR) (13), real-time PCR (14, 15), and particularly 16S DNA sequencing (16). Despite the existing and extensive reports on the prevalence of *P. aeruginosa* in hospital environments, there is still a paucity of research finding on molecular identification of multidrug *P. aeruginosa* strains from surgical wounds particularly in Benin City, Nigeria. Therefore, the present study sought to identify prevailing multidrug *P. aeruginosa* strains from the surgical wound using the 16S rRNA sequencing technique.

## Methods

### Sample Collection

A total of 284 random swab samples of post-operative surgical wound patients (142 from each) were collected from Central Hospital Benin (CHB) and University of Benin Teaching Hospital (UBTH), Benin City.

### Bacteriological Procedures/Identification of Isolates

All samples were aseptically inoculated onto blood, MacConkey, nutrient agar and incubated aerobically at 37 °C for 24 h and checked for colonial growth. Different *P. aeruginosa* strains were isolated from surgical wound samples. They were further identified by using morphological and physiological test (gram staining; oxidase; indole, methyl red, Voges-Proskauer and citrate [IMViC] test; nitrate reduction test and catalase; carbohydrate fermentation test for glucose, maltose, lactose, galactose and sucrose). All specimens were processed at the Lahor Research Laboratories, Benin City, Nigeria using standard microbiological methods. All isolates were identified using conventional techniques as described by Cheesbrough (17).

### Screening Method for Multidrug Resistant P. aeruginosa

Antibiotic screening of *Pseudomonas* spp. isolated from surgical wound swab were carried out with commonly used antibiotic by the Kirby-Bauer disk diffusion method to identify multidrug resistant (MDR) *Pseudomonas* spp. The following antibiotic disks were used: augmentin (AUG 30 μg), ofloxacin (OFL 5 μg), cefixime (CXM 5 μg), gentamycin (GEN 30 μg), cefuroxime (CRX 30 μg), ceftazidime (CAZ 30 μg), ciprofloxacin (CPR 5 μg), nitrofurantion (NIT 300 μg) and interpretation of zones of inhibition according to the Clinical and Laboratory Standards Institute guidelines (18).

### Bacteria Genomic DNA Extraction

All multidrug resistant *P. aeruginosa* isolates were subcultured overnight in Luria-Bertani broth (Merck, Germany) and DNA was extracted from typical colonies of *P. aeruginosa* strains using Zymo research DNA extraction kits (Irvine, CA, USA), according to manufacturer’s instructions.

### Polymerase Chain Reaction Technique

PCR was employed for the amplification of *Pseudomonas* spp. and 16S rRNA primers separately (Table 1) in ABI9700 thermal cycler PCR machine at Lahor Research Laboratories, Benin City, Nigeria. All primers and PCR master mix 2× (New England Biolab, USA) was purchased from lnqaba Biotech, Hartfield, South Africa and was used according to the manufacturer’s instruction. The PCR run was performed in 25 μL reaction mixture containing one part of Quick load (2×) master mix, 1.25 μL of each forward and reverse primer (20 μM), 5.0 μL of nuclease free water and 5 μL of DNA template was added last. The PCR was started immediately as follows: Initial denaturation at 94 °C for 3 min; denaturation at 94 °C for 30 sec; annealing at 50 °C and 54 °C for 30 sec, respectively; extension at 72 °C for 1 min, for 35 cycles; final extension at 72 °C for 10 min; and final holding at 4 °C forever. The amplified PCR products (10 μL) were separated on a 1.0% agarose gel containing ethidium bromide in Tris/Borate/EDTA (TBE) buffer. Electrophoresis was performed at 90 volts for 60 min. Products were visualised in a UV transilluminator and photographed. Amplicon weights were calculated using size maker.

### PCR Product Purification and Sequencing

Amplification and sequencing were done as described by Agbonlahor et al. (21), with the following modifications: Purification was done with the Applied Biosystems Incorporation (ABI) V3.1 Big dye kit according to manufacturer’s instructions. The labeled products were then cleaned with the Zymo Seq clean-up kit (USA) in accordance with manufacturer’s instructions. The ultra-pure DNA was sequenced with ABI3500XL analyser at Functional Bioscience, Madison, USA. Sequences data generated were analysed with Geneious version 9.0.5 and phylogenetic tree were constructed using neighbour-joining method as described by Agbonlahor et al. (21).

### Statistical Analysis

Percentage multiple drug resistance isolates was calculated using the following equation:

$$\%=\frac{\text{Number of MDR isolates from location}}{\text{Total number of isolates from location}}\times \frac{100}{1}$$

## Results

### Biochemical Characterisation of Bacterial Isolates and Distribution of Etiologic Agents of Surgical Wound Infection

Two hundred and eighty-four postoperative wound swabs specimens were collected from patients in CHB and UBTH both in Benin City and analysed. A total of 99 (35%) of patients studied had wound infections. Phenotypic identification of these bacterial isolates using morphological and biochemical tests revealed rod shaped, Gram negative, motile, catalase, oxidase, glucose and citrate positive isolates as well lactose, urease, mannitol, coagulase negative which was suggestive of *Pseudomonas* spp. as shown in Table 2. From both locations, 62 (21.8%) patients (27 from CHB and 35 from UBTH) had *Pseudomonas* spp., 18 (6.3%) patients (8 from CHB and 10 from UBTH) had *Escherichia coli*, 12 (4.2%) patients (5 from CHB and 7 from UBTH) had *Staphylococcus aureus*. *Pseudomonas* spp. is the most isolated pathogen.

### Antibiotic Susceptibility and Resistance

Antibiotic susceptibility profile of all *Pseudomonas* spp. from both locations showed that 17 (63.0%) from CHB and 20 (57.1%) from UBTH had MDR ability against the tested antibiotics. Ceftazidime recorded highest resistance (85.2%) in isolates from CHB while isolates from UBTH showed highest resistance against nitrofuration (77.1%) followed by (68.6%) observed for gentamycin (Table 4).

### Amplification of Pseudomonas spp

**Figure 1:**
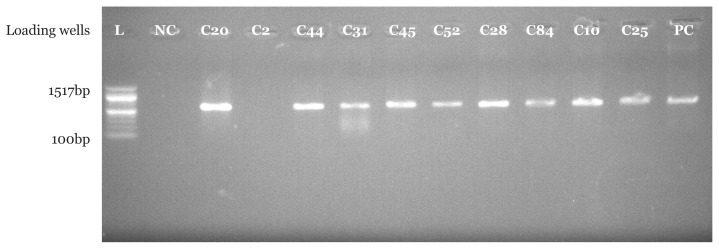
Molecular confirmation of *Pseudomonas* spp. using PCR technique. Isolates C20, C44, C31, C45, C52, C28, C84, C10 and C92 are positive control with bands at 618bp, isolate C2 is not *Pseudomonas* spp. Notes: NC = negative control; PC is a positive control strain with American type culture collection number 27852

**Figure 2:**
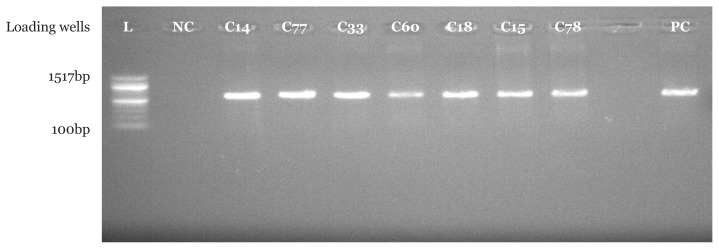
Molecular confirmation of *Pseudomonas* spp. using PCR technique. Isolates C14, C77, C33, C60, C18, C15 and C78 are positive control with bands at 618bp Notes: NC = negative control; PC = positive control strain with American type culture collection number 27852

**Figure 3:**
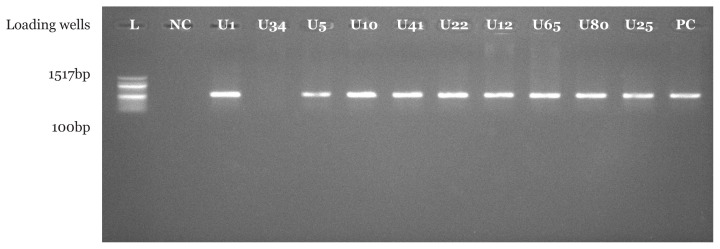
Molecular confirmation of *Pseudomonas* spp. using PCR technique. Isolates U1, U5, U10, U41, U22, U12, U65, U80 and U25 are positive with bands at 618bp. Isolates U34 is negative control for *Pseudomonas* sp. and PC is a positive control strain with American type culture collection number 27852

**Figure 4:**
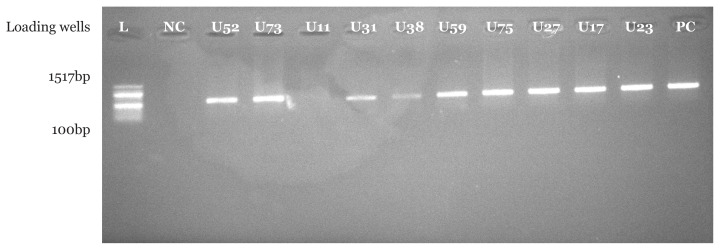
Molecular confirmation of *Pseudomonas* spp. using PCR technique. Isolates U52, U73, U31, U38, U59, U75, U27, U17 and U23 are positive with bands at 618 bp. NC is a negative control; isolates U11 is negative control for *Pseudomonas* spp. and PC is a positive control strain with American type culture collection number 27852

PCR amplification using *Pseudomonas* spp. specific primer set indicated that 16 (94.1%) suspected *Pseudomonas* spp. isolates from CHB and 18 (90.0%) suspected *Pseudomonas* spp. isolates from UBTH were confirmed to be *Pseudomonas* spp. with bands at 618 base pair which were clearly visible under UV transilluminator. In addition, a similar band was also seen for positive control strain with American type culture collection number 27852. As expected, no band was seen in the negative control where nuclease free water was used instead of bacterial DNA as shown in Figures 1–4.

### 16S rRNA Sanger Sequencing of Pseudomonas spp

**Figure 5:**
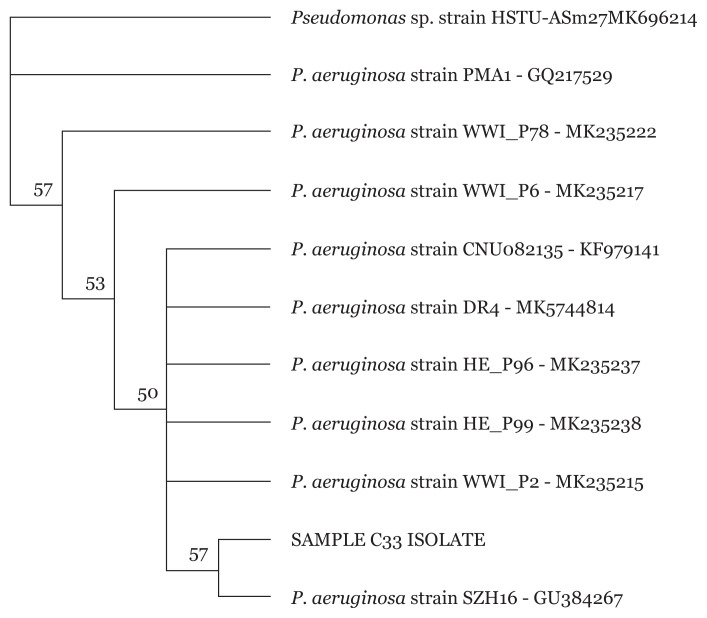
Phylogenetic analysis of clinical isolate based on the nucleotide sequence of part of the 16S rRNA. The phylogenetic tree was constructed by the neighbour-joining method programme in the Geneious package (version 9.0.5). The numbers at the forks show the numbers of occurrences of the repetitive groups to the right out of 100 bootstrap samples. Sample C33 isolate show close relation to NCBI-Blast *P. aeruginosa* strain SZH16 with accession number GU384267

**Figure 6:**
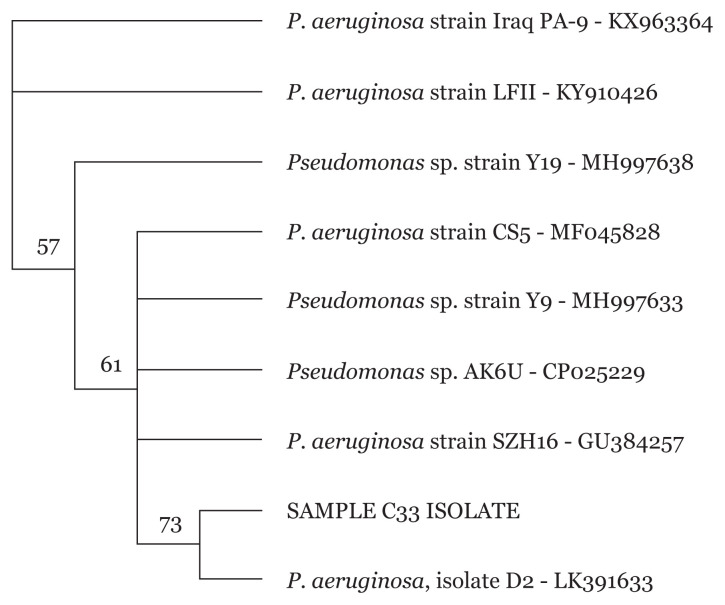
Phylogenetic analysis of clinical isolate based on the nucleotide sequence of part of the 16S rRNA. The phylogenetic tree was constructed by the neighbour-joining method programme in the Geneious package (version 9.0.5). The numbers at the forks show the numbers of occurrences of the repetitive groups to the right out of 100 bootstrap samples. Sample C78 isolate show close relation to NCBI-Blast *P. aeruginosa* isolate D2 with accession number LK391633

**Figure 7:**
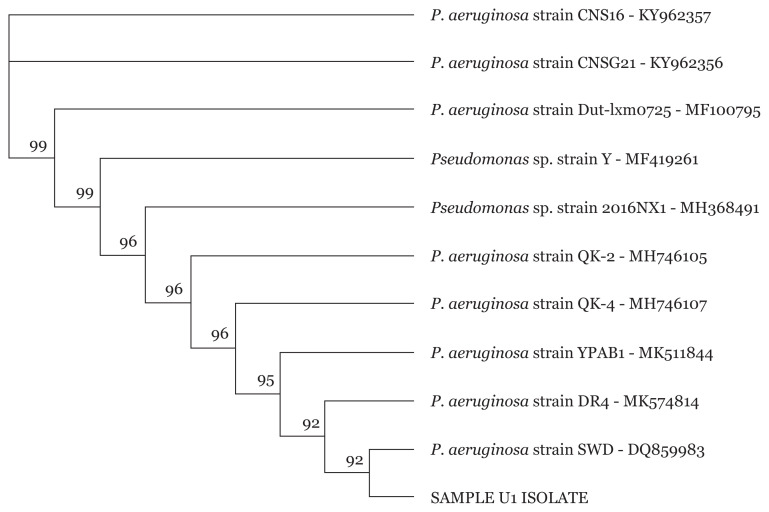
Phylogenetic analysis of clinical isolate based on the nucleotide sequence of part of the 16S rRNA. The phylogenetic tree was constructed by the neighbour-joining method programme in the Geneious package (version 9.0.5). The numbers at the forks show the numbers of occurrences of the repetitive groups to the right out of 100 bootstrap samples. Sample U1 isolate show close relation to NCBI-Blast *P. aeruginosa* strain SWD with accession number DQ859983

**Figure 8:**
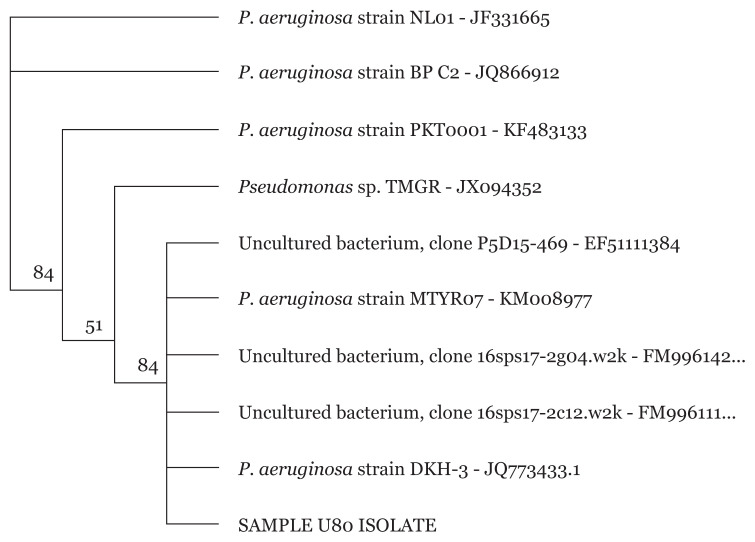
Phylogenetic analysis of clinical isolate based on the nucleotide sequence of part of the 16S rRNA. The phylogenetic tree was constructed by the neighbour-joining method programme in the Geneious package (version 9.0.5). The numbers at the forks show the numbers of occurrences of the repetitive groups to the right out of 100 bootstrap samples. Sample U80 isolate show close relation to NCBI-Blast *P. aeruginosa* strain DKH-3 with accession number JQ773433.1

Sequencing of *Pseudomonas* spp. isolates were carried out to further identify the MDR *Pseudomonas* spp. isolates to the strain level. Phylogenetic tree of isolates revealed different *P. aeruginosa* strains for all 34 *Pseudomonas* spp. as exemplified in Figures 5–8.

### Prevalence of MDR P. aeruginosa Strains from CHB and UBTH

**Figure 9:**
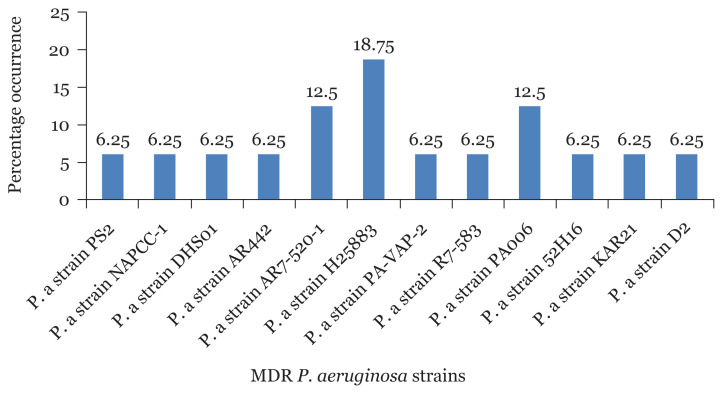
Percentage occurrence of MDR *P. aeruginosa* strains isolated from surgical wound in CHB

**Figure 10:**
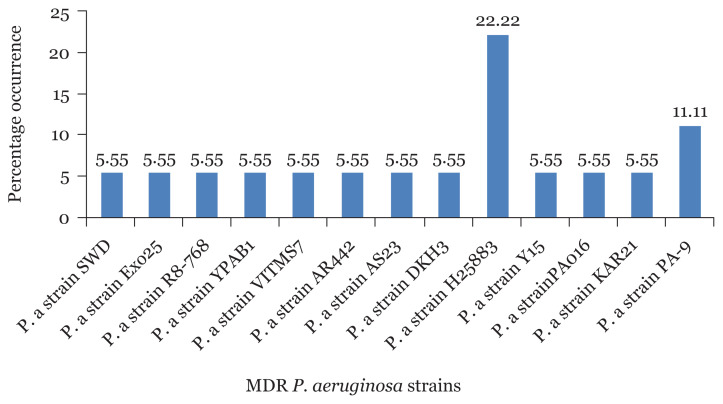
Percentage occurrence of MDR *P. aeruginosa* strains isolated from surgical wound in UBTH

The percentage occurrence of MDR *P. aeruginosa* strains among sequenced isolates from CHB revealed that *P. aeruginosa* strains H25883 had the highest percentage occurrence of 18.75% followed by *P. aeruginosa* strains AR7-520 and PA006 with 12.5%, respectively (Figure 9). In the same vein, *P. aeruginosa* strains H25883 also recorded the highest percentage occurrence of (22.22%) in UBTH followed by *P. aeruginosa* strains KAR21 with 11% as shown in Figure 10.

## Discussion

*P. aeruginosa*, a non-fermentative gram-negative bacterium, is currently the second most widespread nosocomial bacterium, after Acinetobacter species (22). *P. aeruginosa* broadly exists in hospital environments (23) and medical equipment (22). Infections caused by *P. aeruginosa* are particularly tough to treat as the microbe has intrinsic resistance to a large number of antimicrobial agents. Furthermore, with the acquisition of antibiotic-resistant genes, it is becoming more difficult to cure infections caused by this organism (24).

Despite the existing and extensive reports on the prevalence of *P. aeruginosa* in hospital environments, there is still a paucity of research finding on molecular identification of multidrug *P. aeruginosa* strains from surgical wounds, particularly in Benin City, Nigeria. Hence, data from the present study revealed that the *P. aeruginosa* strain showed the highest antibiotic resistance to ceftazidime in isolates from CHB and nitrofurantoin in isolates from the UBTH. Lowest resistance was observed for ciprofloxacin for both locations. In comparison to previously reported data, the result of the present study corroborates the finding of Carroll et al. (25) and Leone et al. (26) that also reported a high antibiotic resistance rate towards ceftazidime and gentamycin antibiotics in both clinical and environmental isolates. Additionally, the study of Ruiz et al. (27) reported that clinical bacterial isolates are less susceptible to antimicrobial agents than environmental bacterial isolates due to their selective action (27).

Molecular characterisation of *Pseudomonas* spp. isolated from surgical wound infections specimens from both locations showed that 16 (94.1%) out of 17 isolates were confirmed to be *Pseudomonas* spp. in CHB, while 18 (90%) out of 20 were confirmed to be *Pseudomonas* spp. with bands at 618 base pair for test isolates and positive control strains. The above-mentioned genus-specific PCR assays indicated that three clinical isolates had been misidentified using phenotypic laboratory methods. This signifies the efficiency of the molecular characterisation method over phenotypic characterisation. With regard to a study by Spilker et al. (19), the genus-specific PCR assays indicated that several of the 66 clinical isolates were misidentified by the referring laboratories (19).

Sequence analysis of 16S rRNA is now being used as a taxonomic ‘gold standard’ in determining the phylogenies of bacterial species (28). The 16S rRNA gene sequences comprise hypervariable regions with high conservation that can differentiate species-specific signature sequences helpful in the classification of bacteria (29, 30). Going forward, 34 (91.9%) *Pseudomonas* spp. were further examined by 16S rRNA sequence analysis, and in each case, the PCR assay results were consistent. Thus, when this set of isolates was assessed against the 16S rRNA sequence, the sensitivity and specificity of both PCR assays were again 100%. It has also been reported that selective amplification of *Pseudomonas* 16S rRNA analysis is used to detect and differentiate *Pseudomonas* species from clinical and environmental samples (30). The present results agree with the finding of Didelot et al. (32) who reported that 16S rRNA gene sequencing is now common in medical microbiology as a quick and inexpensive alternative to phenotypic approaches of bacterial identification.

The result from the phylogenetic trees showed that MDR *P. aeruginosa* strain H25883 was the predominant strain in both locations (CHB and UBTH) with 18.75% and 22.22%, respectively. *P. aeruginosa* strains AR7-520 and PA006 with 12.5% were observed in CHB, making it the second predominant strains. However, many studies reported that *P. aeruginosa* has been mostly isolated from post-operative surgical wounds regardless of the site of infection and location of samples as a result of its high survival uniqueness in the hospital setting (33, 34). It has been ranked second among nosocomial disease-causing microbes. They are isolated from hospitals frequently, contaminating hospital equipment such as sinks used for wound dressing and other surgical tools. Furthermore, many antimicrobial-resistant strains continue to exist in apparently sterile hospital equipment, therefore, making it a precarious nosocomial pathogen broadly dispersed in the hospital environments where they are most difficult to eliminate (35).

The 16S rRNA sequence analysis on all identified MDR isolates was carried out and the sequence data confirmed the PCR results. Though our PCR and DNA sequence analyses showed bacterial isolates that were misidentified by phenotypic testing, we must point out that this study was not intended to determine the incidence of misidentification of post-surgical wound bacterial isolates neither to evaluate the relative precision of different phenotypic identification systems. Both assays have 100% sensitivity and specificity for their intended targets. We have also established the utility of these PCR assays in precisely identifying *P. aeruginosa* strains among isolates not correctly identified by phenotypic analyses. These assays should serve as a valuable accessory in the evaluation of gram-negative non-fermenting bacteria recovered from surgical wound isolates.

## Conclusion

The results obtained from our study revealed that the 16S rRNA-based PCR and sequencing are highly sensitive, precise and consistent for the identification of *P. aeruginosa* strains isolated from post-operative surgical wound infections than conventional bacterial phenotypic methods. Our finding further highlights the use of DNA sequencing of the 16S rRNA gene as an effective tool to study bacterial phylogeny and taxonomy associations between bacteria and bacterial detection as well. Thus, early identification and control of this pathogen have become increasingly important.

## Figures and Tables

**Table 1:** Sequence information of primer used

S/N	Primer name	Primer sequence (5′ to 3′)	Target gene or region (s)	Product length (bp)	Reference
1	PA-GS-F PA-GS-R	GACGGGTGAGTAATGCCTA CACTGGTGTTCCTTCCTATA	*Pseudomonas spp*.	618	(19)
2	27F 1492R	AGAGTTTGATCMTGGCTCAG CGGTTACCTTGTTACGACTT	16S rRNA	1500	(20)

**Table 2:** Morphological and biochemical characterisation of bacterial isolates

Characterisation	Group A	Group B	Group C	Group D
Shape	Rods	Rods	Rods	Cocci
Gram’s staining	−ve	−ve	−ve	+ve
Motility	Motile	Motile	Motile	Non- motile
Catalase	+ve	+ve	+ve	+ve
Oxidase	−ve	+ve	−ve	−ve
Glucose	+ve	+ve	+ve	+ve
Sucrose	−ve	−ve	−ve	+ve
Maltose	+ve	−ve	−ve	+ve
Lactose	+v	−ve	−ve	+ve
Oxidation fermentation	Fermenter	Oxidiser	Facultative anaerobes	Fermenter
Mannitol	+ve	−ve	−ve	+ve
Urease	−ve	−ve	+ve	+ve
Citrate	−ve	+ve	−ve	+ve
Nirate	+ve	+ve	+ve	+ve
Indole	+ve	−ve	−ve	−ve
Methyl red	+ve	−ve	+ve	−ve
Coagulase	−ve	−ve	−ve	+ve
Bacteria suspected	*Escherichia coli*	*Pseudomonas* spp.	*Proteus mirabilis*	*Staphylococcus aureus*

Notes: +ve = positive; −ve = negative; Group A, B, C, D = different isolates

**Table 3:** Distribution of etiologic agents of surgical wound infection

S/N	Bacterial isolates	UBTH	CHB
1	*Pseudomonas* spp.	35	27
2	*Escherichia coli*	8	10
3	*Proteus mirabilis*	4	3
4	*Staphylococcus aureus*	7	5

	Total	54	45

**Table 4:** Susceptibility profile of suspected *Pseudomonas* spp. isolates to tested antibiotics

Class of antibiotics	Type of antibiotics	CHB *n* = 27		UBTH *n* = 35	
	
		R	S	R	S
Penicillin	Augmentin (30 μg)	17	10	22	13
Aminoglycoside	Gentamycin (30 μg)	19	8	24	11
Cephalosporin	Ceftazidime (30 μg)	23	4	21	14
	Cefuroxime (30 μg)	19	8	20	15
	Cefixime (5 μg)	22	5	20	15
Nitrofuran	Nitrofuration (300 μg)	18	9	27	8
Quinolones	Ofloxacin (5 μg)	16	11	18	17
	Ciprofloxacin (5 μg)	13	14	13	22

Notes: *n* = number of bacteria tested; R = number of bacteria resistant; S = number of bacteria sensitive
